# Public Health Risks in Urban Slums: Findings of the Qualitative ‘Healthy Kitchens Healthy Cities’ Study in Kathmandu, Nepal

**DOI:** 10.1371/journal.pone.0163798

**Published:** 2016-09-29

**Authors:** Helen Elsey, Shraddha Manandah, Dilip Sah, Sudeepa Khanal, Frances MacGuire, Rebecca King, Hilary Wallace, Sushil Chandra Baral

**Affiliations:** 1 Nuffield Centre for International Health and Development (NCIHD), University of Leeds, Charles Thackrah Building, Leeds, United Kingdom; 2 Health Research and Social Development Forum (HERD), Kathmandu, Nepal; 3 Burn Injury Research Unit, The University of Western Australia, Perth, Australia; Australian National University, AUSTRALIA

## Abstract

**Background:**

Communities in urban slums face multiple risks to their health. These are shaped by intermediary and structural determinants. Gaining a clear understanding of these determinants is a prerequisite for developing interventions to reduce the health consequences of urban poverty. With 828 million people living in slum conditions, the need to find ways to reduce risks to health has never been greater. In many low income settings, the kitchen is the epicentre of activities and behaviours which either undermine or enhance health.

**Methods:**

We used qualitative methods of semi-structured interviews, observation and participatory workshops in two slum areas in Kathmandu, Nepal to gain women’s perspectives on the health risks they faced in and around their kitchens. Twenty one women were interviewed and four participatory workshops with a total of 69 women were held. The women took photographs of their kitchens to trigger discussions.

**Findings:**

The main health conditions identified by the women were respiratory disease, gastrointestinal disease and burn injuries. Women clearly understood intermediary (psychosocial, material and behavioural) determinants to these health conditions such as poor ventilation, cooking on open fires, over-crowding, lack of adequate child supervision. Women articulated the stress they experienced and clearly linked this to health conditions such as heart disease and uptake of smoking. They were also able to identify protective factors, particularly social capital. Subsequent analysis highlighted how female headed-households and those with disabilities had to contend with greater risks to health.

**Conclusions:**

Women living in slums are very aware of the intermediary determinants–material, behavioural and psycho-social, that increase their vulnerability to ill health. They are also able to identify protective factors, particularly social capital. It is only by understanding the determinants at all levels, not just the behavioural, that we will be able to identify appropriate interventions.

## Introduction

Globally, it is estimated that 828 million people live in slum conditions, representing around one third of the world’s urban population. This figure is set to grow, particularly in South Asia with urban populations projected to rise from 45% to 62% by 2050 [1]. Attention to the health inequalities between slum and non-slum areas is important to ensure health outcomes for the urban poor maintain the so-called urban advantage [2]. We aimed to develop an intervention to address public health risks facing slum dwellers in Kathmandu, Nepal. Nepal is experiencing the most rapid rates of urbanisation in South Asia with urban population growth rates of up to 7% per annum [3]. In order to inform this intervention, we sought first to identify, from the perspective of women living in slums, environmental hazards present in the kitchen and the wider slum environment and their associated health risks. We also sought to explore their understanding of the determinants of their increased exposure to these hazards.

Global data clearly illustrate the impacts of living in hazardous environments within the home. Unimproved water and sanitation is ranked second highest in contributing to disability adjusted life years in LMICs, causing 60 million DALYS globally (gastro-intestinal disease)[4,5]; use of solid fuels for cooking and heating ranks 5^th^ highest, causing over 40 million DALYS [4]and 3.5 million premature deaths annually (respiratory disease), particularly affecting women and children [6]. Injuries are an important and often forgotten cause of mortality and disability in South Asia where over 30% of global unintentional injury deaths occur [7]. After road traffic accidents, falls, drowning and burns are the most common cause of death from injury [7]. Most burns in South Asia occur in the home, with women over 14 and boys under 12 at most risk [8].

Data on slum living conditions in Nepal is limited and linking these risk factors to health outcomes is challenging as national household surveys rarely contain sufficient samples of the urban poorest [9]. One of the few surveys of slum communities in Kathmandu found 98% of households living in semi-permanent or temporary accommodation, 15% practiced open defecation and 48% used latrines that discharge directly into a river [10]. For cooking, about 40% use firewood, 20% kerosene and the remainder use gas (LPG) stoves [10]. In a smaller survey of Kathmandu slums, 7% of household members suffered a major health problem during the year preceding the survey, with gastro-intestinal disease, respiratory disease, accidents and injuries among the main causes [11].

Health interventions frequently focus on one condition or risk factor; however, in slum households multiple risk factors coalesce to contribute to the high burden of morbidity and mortality. The kitchen is the epicentre of activities and behaviours that either enhance or undermine health. In order to develop an appropriate intervention and understand potential barriers to behaviour change, we felt it important to explore what women know about the risks to their families’ health related to the kitchen and the wider slum environment. To understand the wider social determinants influencing the health of slum communities we used the Commission for the Social Determinants of Health framework [12,13] to identify structural determinants of health inequalities and intermediary determinants, or ‘circumstances of daily life’, that result in differential vulnerability to disease-causing influences.

## Methods

### Setting

Data was collected during a six-month period from May 2013 to October 2013 in two slum settlements in Kathmandu. Slum A is situated in the south east of Kathmandu metropolitan city on the banks of the Manohara and Hanumante rivers and is one of the oldest slums in the city. Slum B is a more recent development on the banks of the Bagmati river. While there is a lack of specific data on the conditions in each slum, Slum A appears to have better economic conditions, with a greater proportion of cement block and brick built homes than Slum B. The communities in both slums are squatters with no legal rights to their land. While slums in Kathmandu are by no means homogenous, these two slums were selected as they illustrate different characteristics, with one being long established with better-off residents and the other being a more recent development with poorer residents.

### Participants

As women are predominantly responsible for the domestic sphere in Nepal, they were the population for this study and purposively sampled to ensure a spread of caste and ethnic background, age and socio-economic status. The caste system. While approximately 80% of the population are Hindu, the caste system has been, and is still, a major determinant of identity, social status and life chances of all Nepalese [14]. The main caste groups are Brahaman/Chhetri, Tarai/Madhesi Other Castes, Dalits, Newar, Janajati/Adivasi. Dalits–and especially Dalits from the Tarai–Muslims, and Tarai/Madhesi Other Castes have been found to have consistently low lower social and health outcomes, followed closely by the Janajati/Adivasi [14].

The two research organisations implementing the study in Kathmandu have been running primary health care clinics serving the two communities for a number of years. This has enabled them to build up a detailed knowledge of the communities. The health workers in each of the clinics worked with the research team to purposively sample women to provide the variation of experiences and views needed.

A team of five Nepalese researchers from these two NGOs conducted the field work; four were educated to degree level and the one senior researcher, to MSc level (author initials). The team consisted of two female and three male researchers. As interview topics were not of a sensitive nature, the use of male interviewers with female participants was not felt to be a particular limitation. The team had varying levels of experience in qualitative research. A more experienced team members was coupled with a less experienced researcher to provide support and guidance. The team were overseen by the PI (first author) who is an experienced qualitative researcher. The PI provided a one-day training session for all of the researchers on qualitative and participatory approaches. The women were approached at their homes by two researchers who verbally explained the information sheet. Participants were given the opportunity to answer questions and consider their involvement before consenting. This approach was used to recruit participants to both the qualitative interviews and the participatory workshops.

### Data collection

Three qualitative methods were used to collect data: semi-structured interviews, observation and participatory workshops. The researchers conducted all interviews in pairs with women in their homes and all five researchers helped to facilitate the participatory workshops. The interviews were guided by a list of open-ended questions; however, the interviewers also probed on particular areas of interests to the women and relevant to the topic. Topics included demographic information, kitchen practices particularly cooking, eating, washing, heating, lighting, health problems and women’s understanding of their causes.

Our previous experience has highlighted the challenges in encouraging women to talk freely and openly during interviews. Visual methods are a well-established approach to data generation in qualitative research[15,16], particularly to provide insights on social, cultural and contextual factors [17]. Photovoice [18], where participants are encouraged to take photographs of themes important to them, was felt to be particularly suitable to stimulate discussion during interviews in this study. The women were given cameras a few hours or days before the interview and asked to take photos of key aspects of their kitchens and homes. The researchers then used these to stimulate discussion during the interview. With the women’s consent, all photographs and diagrams were used within the analysis.

The researchers made observations during the interviews, noting down details of the number of rooms, their use, housing materials, ventilation, type of stove, fuel use, food storage, availability of any kitchen equipment, location and state of washing and sanitary facilities and any other observations about the kitchen, home or household members. The observation notes were attached to the interview transcripts and used within the analysis.

Four participatory workshops were planned, two in each slum. The objectives of the workshop were to facilitate the women to analyse as a group the risks to health in slum kitchens, any seasonal variation and links to common health problems. The women then built on this critical analysis to identify solutions that they themselves or external organisations could implement to reduce health hazards. Stimulating in-depth group discussions can be challenging, particularly in the context of different caste, wealth and education backgrounds among the women. In order to overcome these hierarchical divisions, participatory methods [19,20] were used with smaller groups within the workshops. Specific methods included ‘But why? Exercise’, seasonal calendars and ranking matrices [19].

Audio recording of the simultaneous group discussions during the workshops proved problematic, both due to audio quality and challenges in identifying participants. In light of this, researchers took detailed notes of the group and plenary discussions. All diagrams were photographed and the researchers recreated these typed documents for use in the analysis.

The researchers kept reflective notes following each interview and following the facilitation of the workshops. They recorded any impressions they had participants’ responses to questions and interacted with researchers. Following the workshops, researchers reflected on the interaction between participants and their engagement with the topic, how well the participatory exercises had gone and how facilitation could be improved. The notes were included in the analysis.

The Framework Approach [21] was used to analyse the data. This five step process provides a relatively deductive way to analyse qualitative data which can be structured around a pre-determined framework of key themes whilst also allowing for new themes to emerge from the data. This approach enabled the analysis to stay focused on public health risks and perceptions of their impact on health with the framework of the CSDH. All audio recordings were translated into English as they were being typed by the Nepalese researchers. The typed transcripts were then read through by two of the researchers and any areas where the translation was not clear were discussed with the researchers who had conducted the interviews. The initial coding was done by (2x author initials) and the thematic framework was developed in discussion with the (3x author initials) based on the original research questions and the themes emerging from the data. Nvivo 9 was used to assist the organisation of the data and the analysis process. Details of the methods used and the data sources they produced are presented in Table 1.

**Table 1:** Summary of data sources.

Method	Data used in the analysis
Individual interview	21 transcripts
Observation	Observation notes from researchers from 21 interviews Subsequent analysis of photos from 19 of the 21 participants
Participatory workshop	Notes from the 4 workshops; seasonal calendars; ‘But Why?’ diagrams of kitchen risks; ranking matrices of interventions
Reflection	Reflective notes following each interview (21) and workshop (4)

The study received ethical approval from the Nepal Health Research Council and from the University of Leeds Ethical Review Committee. The use of cameras was discussed with the women during the process of gaining informed consent which was taken by signature or by thumb print depending on literacy levels. Women were informed that if their photos included other people then the consent of these individuals should be sought for their inclusion in the study. A section of the consent form enabled the process of gaining the consent of these participants. If people did not agree to their image being used within the study, these photos were to be deleted from the digital cameras during the interviews and not used in any data analysis. In practice, all those interviewed and photographed agreed to the use of their photos in the study.

## Results

Twenty-one women were interviewed in total, 11 in slum A and 10 in slum B. Interviews lasted between half an hour and one hour and were all conducted in the women’s homes. The median age of the women was 40 years old with a maximum of 70 years and a minimum of 30 years. The women had a median of two children living at home, with the maximum being 6 children and four women without any children currently at home. We had wanted to collect information on each woman’s caste and ethnicity, however 11 women did not share this information, of the remaining women interviewed, four self-identified as Aadibasi, one as Tamang, two as Chhetri, two as Dalit and one as Newar. Demographic details of all participants can be found in Table 2.

**Table 2:** Characteristics of the women interviewed.

Interview code	Age	Caste	Better off/Poor/ Very Poor	Children living at home	Work	Marital status M = marriedS = single	Researchers’ Observations
M001	34	Not provided	Poor	2	no work	M	She lives with her husband, but both husband and wife are too ill to work. They have no land, property or source of income. The house was bought for them by her maternal family. The house is made of cement blocks, with four rooms: two bedrooms, a kitchen and a living room. They have a well-managed kitchen with a gas-stove.
M002	38	Not provided	Poor	2	works	M	Her husband gets occasional labour work and she rears pigs. She uses a gas-stove and as wood stove when she cannot afford a gas cylinder. The house is made of brick with a tin roof.
M003	70	Not provided	Poor	None	husband works	M	Her husband is a daily wage labourer. They have no children. Whatever they earn they spend it on treatments and fuel. They use a wood stove and borrow money to buy fuel. They often cannot afford a proper diet (milk and curd).
M004	53	Not provided	Poor	2	no work	S	She lives with her daughter and not her husband. Her monthly income is Rs. 2000 and she has no savings. The house is brick with a tin roof. Her daughter is taking beauty parlour training.
M005	50	Not provided	Better off	None	works	S	She works as a peon in a government ministry where she earns Rs. 8000 per month. She is not with her husband. Family members stay with her and earn income too. They can afford fresh food including vegetables, a battery chargeable light and a gas stove. Their house is well built with a tin roof.
M006	42	Not provided	Poor	3	husband works	M	Her husband is the sole bread-winner, he drives a taxi. They struggle financially as they have three children to support. They have a small two roomed small house accommodating 5 people.
M007	42	Dalit	Poor	3	husband works	M	She has no job, but her husband works as a labourer. They use a saw-dust stove and cannot afford vegetables, fruits and pulses and cannot afford electricity. They eat rice with pickle.
M008	35	Adivasi	Poor	3	husband works	M	Her husband and sometimes the children work as daily wage labourers. They cannot afford gas and only occasionally buy fruit and meat. They have electricity, but use candles during load-shedding. They have one room for five people.
M009	30	Dalit	Poor	None	works	S	She lives alone as she was abandoned by husband. She has no children. She works as a wage labourer, earning just enough to live day-by-day. She has a firewood stove and no toilet. She has no savings.
M010	30	Tarai Chhetri	Poor	2	works	S	She has a job in a canteen where she earns Rs. 4000 per month. From this she supports two children and her mother. She uses a wood stove and candles during load-shedding when she can afford them.
M011	55	Tamang	Poor	1	works	S	She lives alone and works as a daily wage labourer in construction where she earns Rs. 350 per day. She normally works 15 days in a month. She has no toilet. Previously she used a wood stove, but son-in-law bought her a gas-stove a few months ago. She could not afford to educate her daughter.
S001	50	Not provided	Very poor	1	husband begs	M	She is disabled and only her husband can earn for the family. He sometimes begs at the temple, but it is difficult to feed the family and manage the home.
S002	40	Not provided	Very poor	6	husband works	M	Her husband is a traditional healer, but only earns money irregularly. She used to wash dishes but they paid so little. It is very difficult to manage 6 children and they had to take a loan.
S003	56	Not provided	Very poor	1	works	S	Her husband has died, so she is alone. She does embroidery designs for pashminas, earning Rs. 30 from one shawl. Her home is in a very poor state. No toilet. Wood stove.
S004	60	Not provided	Very poor	None	no work	S	She has no sources of income and can no longer work. She gets some senior citizen incentives but has no other source of income. Her home is poorly constructed. She uses firewood for cooking and lives in a single room. She is clearly very poor.
S005	40	Not provided	Poor	3	works	M	She is married and works as a labour. She says she is able to manage the household and care for her three children. The kitchen and house are very basic.
S006	44	Newar	Very poor	3	husband works	M	The house was of very poor construction, with no door to lock. Security and safety were a concern. They are clearly very poor.
S007	36	Chhetri	Poor	2	no work	M	The house was of a fair standard with cement brick walls and tin roof. They had two rooms, one with a big window for ventilation. The roof was made of tin and the wall of cement bricks. They had a TV and the kitchen was organised.
S008	30	Aadivasi	Poor	3	no work	S	She was living alone without her husband. She struggles to manage everything by herself, although she is supported by her maternal household who bought this home for her.
S009	30	Aadivasi	Poor	3	husband works	M	Her husband works as a labourer and finds it difficult to manage financially. They live in a brick house with a tin roof, but the walls are low and not plastered.
S0010	40	Aadivasi	Better off	2	works	M	She has pigs and has been able to make of good profit. She also sells alcohol for domestic use. Her house is well built with cement blocks and a tin roof, which she can collect water from when it rains.

Nine women in slum A and 10 in slum B agreed to take photographs of their kitchens for the study. The researchers’ reflective notes highlight how the women who did take photos enjoyed this process and how discussing the images brought lightness and humour into the discussions. It is also evident that the interviews with women who did take photos are longer and more detailed. As one researcher explained:

“At first she was hesitating to talk with us, but as time went on she told us everything without any hesitation. She was happy to take the photos with our camera and we discussed the picture in such an interesting way. She told us that this was the first time that she had taken a photo in her life. Using the camera made the interview much more interesting”

(Research Assistant’s reflective notes).

Four participatory workshops were held. Two in slum A, one with 19 participants and other with 15 participants, and two in slum B with 17 and 18 participants respectively. The workshops were all held in community venues and lasted between 2 and 3 hours. The women divided themselves into groups for discussion during the workshop. During the first exercise each woman drew a map of her own kitchen and described the key features to the others in her group. This stimulated discussion on kitchen equipment and practices. In the second activity the group identified risks in their kitchen and used the ‘but why?’ technique to identify their underlying causes. The women then constructed a seasonal calendar to identify seasonal variation in the risks and health problems they and their families experienced. The participants all agreed to categorise the seasons as garmi (hot/summer), barkha (wet/monsoon) and jado (cold/winter).

The participants in slum B were less eager to participate in the workshop than those in slum A. A limitation of the study was that there was no means of providing child care for the women during the workshop. While this does not seem to have been a constraint in slum A, in slum B the women struggled to participate fully as they simultaneously cared for their children. The researchers’ reflections conclude that lower literacy levels in slum B meant participants required more support from the researchers and took longer to understand the exercises. The use of participatory exercises in this context was new, both to the participants and the facilitators. However, the exercises played a key role in generating discussions and analysis of kitchen-related risks, their causes and possible solutions.

“At first they were a bit puzzled, and didn’t know how to start, but when the facilitators helped, they got interested [in the exercises] and began to speak up more.”

(Researcher facilitator, participatory workshop in Slum B)

**Fig 1:**
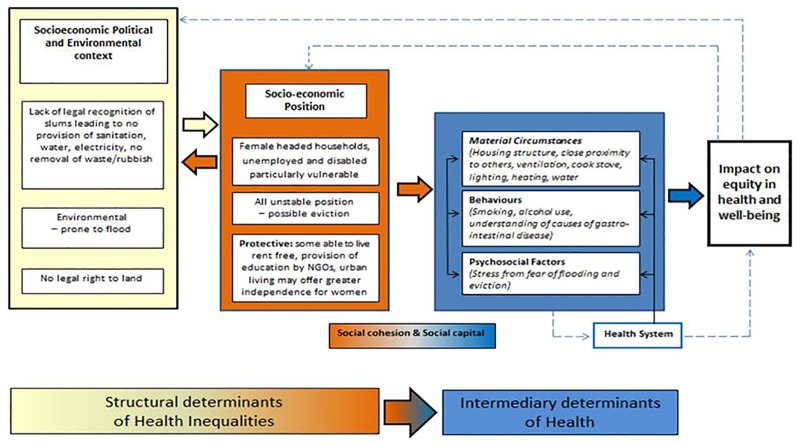
Social Determinants of Health in Kathmandu Slums. The CSDH [12] framework depicting the social determinants of health has been used to group the key findings of the study into structural and intermediary determinants of health inequalities resulting in differential vulnerability to and protection from communicable and non-communicable diseases.

The specific household health issues that women raised and their perceptions of the determinants influencing the risk of suffering these health concerns are presented below. In Fig 1, these factors are depicted within the framework of the CSDH. The seasonal dimensions to these risks are also highlighted. Finally, the women’s recommendations for interventions that they themselves could take forward and those that other institutions could work on are presented.

### Health Issues Identified by the Women

The most commonly identified health complaint by the women was respiratory ill-health, particularly asthma, cough and tuberculosis. This was closely followed by complaints of gastro-intestinal disease. Many women also identified heart disease and stress as important health concerns. The women mentioned, although to a lesser extent, burn injuries, skin and eye irritation and headaches. The causes and wider determinants of these conditions that were identified by the women are presented below.

#### Respiratory Health

Smoke from stoves and fires for cooking and heating was clearly seen by the women as a cause of their respiratory complaints.

“Because of smoke, I suffer from eye-irritation, the baby cannot sleep properly. There are numerous other internal effects as well…. If the wood isn't dry, then a lot of smoke is emitted…..I experience a burning sensation in the eyes. I need to blow into the fire continuously till it burns. It gives me a headache and cough.”

(S007: 36 year old woman, poor, married with two children and no work)

“Smoke enters with the breath and then it affects us a lot, it also causes asthma… I heard about this from friends and on the radio.”

(SO09: 30 year old woman, poor, with three children and a husband with work)

The women also recognised how the structure of their homes contributed to their exposure to smoke. The poorer homes had little ventilation and cooking was done in cramped conditions. The two slums were by no means homogeneous in terms of poverty levels. Levels of wealth–a structural determinant of health—clearly determined the type of fuel used and the structure of the home–intermediary determinants of health. Within the two slums, the researchers observed several different forms of stove and fuel use. Better off households had two-burner gas stoves connected to an LPG canister, while others had clay stoves using wood, sawdust or charcoal. Women explained that wood and sawdust were the cheapest form of fuel.

“Most have gas. In this neighbourhood only I and a few other houses have a chula [firewood stove] in their house. I can't use gas because it is more expensive than firewood. Two cylinders are also not enough for a month for me to cook the meals. I have no income, so how I can buy gas?”

(M008: 35 year old woman, poor, with three children and a working husband)

During the participatory workshop, the women ranked types of stove against the criteria of cost, fuel availability, efficiency and safety. Use of biogas was ranked lowest as, while it was cheap, availability of animal dung was too minimal to make it a viable option; improved cook stoves were seen as a strong option, but there was much debate on whether these stoves, while heavily promoted in rural areas might be unavailable in urban areas. Overall the women ranked gas as the most efficient and safe means of cooking. For the women owning a gas stove was an aspiration, even for the poorest households who knew they were too expensive to buy and use.

While recognising the negative health impacts of using wood, many women with gas also used wood for specific cooking needs, such as making the local alcohol, Raksi. The wood stove was vital during times of short supply of gas or when household income was strained. With average winter temperatures in Kathmandu of 2°C, households were quick to explain how during the cold months wood fires were a necessity for warmth.

“Most of the times, we use gas. But in the winter, the cold is torturing so we sometimes opt for firewood. We use that clay stove (points to a clay stove under the table). It keeps us warms as well.

(M001: 34 year old woman, poor, married with two children and no work)

For the very poorest, even occasional fires for warmth during the winter months were unaffordable:

“We wear woollen clothes; it's costly to use firewood so, we don't use fire.”

(S004: 60 year old woman, very poor, single, no children at home and no work)

Several of the poorer women explained how they would build communal fires, acting as a catalyst for a social gathering as well as a necessity to keep warm:

“I keep warm with the clothes I get while begging and sometimes we build a fire outside and gather around it with relatives.”

(S001: 50 year old woman, very poor, one child and a husband who must beg for money)

It was not only the material circumstances with the household that determined poor respiratory health; the circumstances and close proximity of neighbours also determined exposure to smoke, with smoke from one household inevitably entering neighbouring homes, as one such firewood user confesses:

“Yes, we have a wood stove and there is a lot of smoke. It escapes out through the holes. It goes into the neighbours houses too. They complain audibly and wonder aloud where the smoke is coming from. We all remain quiet!”

(M002: 38 year old woman, poor, married with two children and working)

#### Gastro-intestinal disease

The majority of participants noted the frequent occurrence of diarrhoea and gastritis. There was a common perception that stomach problems were caused by eating foods that were not ‘good and fresh’:

“I think the fact that we do not get good food in time to eat, we cannot eat foods like milk and fruits. These are the reasons behind these stomach problems. Fresh and good food costs more than stale food. So, we opt for the stale food.”

(S006: 44 year old woman, very poor, with three children and a husband who works)

During the participatory workshops, the women clearly identified seasonal dimensions to outbreaks of diarrhoea, with the increase in flies during hot summer (Garmi). Many identified challenges with storing food as they had no proper containers or place to keep supplies. Keeping food and belongings dry and clean during the monsoon season (Barkha) was also identified as a constraint to keep food fresh and clean.

While several women were able to reiterate health promotion messages on the importance of boiling water, few actually associated their lack of access to clean water or sanitation as a risk for gastro-intestinal disease. This was despite the fact that both slums faced considerable challenges in accessing clean water particularly during the winter months. In both slums access to water was either at a government supplied tap or from a tanker delivering water. Neither of these sources were seen as clean or readily available:

“We bring water from the government tap. It's not difficult to bring water but the water is not clean. The water is only available two or three times a week; we have to stand in line to get water. Yes, we use the same water for cooking and washing.”

(S001: 50 year old woman, very poor, one child and a husband who must beg for money)

A covered storage jar full of water was a common feature in most kitchens. The poorest used this water untreated.

“No, we do not do anything [to the water]. We use water from the jar directly.”

(S007: 36 year old woman, poor, married with two children and no work)

Those that did mention boiling water were frequently those that also mentioned being exposed to NGO education campaigns and were also those that were better off. It was unclear whether this was due to a lack of education about the need for clean water, or the cost of fuel needed to boil water. Either way, the poorest households were restricted in their means of access to clean water.

#### Accidents and Burn Injuries

The majority of women had experienced some form of minor accident or burn injury. Several immediate determinants were considered to influence the occurrence of injuries: in particular lack of storage areas for sharp implements, the use of stoves–gas or wood–in cramped conditions; needing to combine child care with cooking and huddling round fires for warmth. This woman explains how easy it is for a child to get burnt when the mother is cooking and caring for children simultaneously:

“My younger daughter…. was clinging on to me and trying to grab my hands on one side and I was holding the pan with the food with my hands on the other side. She was using her full force to pull me down; I was trying to hold the pan upright and also trying to prevent the baby from falling. I couldn't balance the food and the baby; the food fell from my hands and over my daughter's face. The food was really hot. But we were lucky because my daughter was fully dressed so she was only burned a little.”

(M001: 34 year old woman, poor, married with two children and no work)

The photos used by the women in the interviews clearly illustrate the challenges they faced trying to manage their household within a very small space.

“That is the cooker and this is firewood (pointing to the photo). I have no separate room or space, so I cook meals on my bed but it is really not good for us, because it may cause burns and any little mistake could turn my home to ashes. It has also caused my mother to develop asthma. But what can I do? I don’t have enough space.”

(M010: 30 year old woman, poor, single with two children and working)

#### Stress

While stress was not a factor covered in the initial interview guide, it emerged as an important health concern during the early interviews and was explored in subsequent interviews. Many women associated the levels of stress they experienced with subsequent hypertension and high levels of smoking. There were two predominant structural determinants of stress identified by the women in both slums: the risk of flood and threat of eviction. Informal settlements spring up on the cheapest and most undesirable land in Kathmandu; this is inevitably along the banks of the main rivers. These rivers are heavily polluted with the city’s rubbish. During monsoon season the rivers present even greater risks as they are prone to flooding.

“Six years ago on the day of Krishna Janma Aasthami [Hindu festival in August] my daughter was carried away by the flood. She was taken out of the river after sometime and taken to hospital. At that time, I felt great tension. After a while I started smoking.”

(SO10: 40 year old woman, better off with two children, married and working)

The continual fear of flood during the monsoon season was expressed in both the slum A and B.

“It is very difficult for us during times of flood. We have to live in a state of fear; we fear the flood is going to take away the houses and harm people living here.”

(S002: 40 year old woman, very poor, with six children and a husband who works)

Due to their illegal status, both slum communities also faced the continual fear of eviction:

“The bad aspect is that the government is constantly threatening to evict us which is the reason why I'm constantly worried. I have to go to rallies and andolans [rebellions]. We don't know when we are going to be forcefully evicted.… It is difficult for us to sleep peacefully at night.”

(M001: 34 year old woman, poor, married with two children and no work)

“We are afraid of the government because the police can come and tell us to evacuate this place any time. They can also use force so we are always in fear and under a lot of stress.”

(S006: 44 year old woman, very poor, with three children and a husband who works)

### Community assets and social capital

An important determinant that emerged from the analysis was that of social capital and the role of social cohesion in their health and well-being. Several women identified the importance of the relationships they had developed with neighbours and the local community.

“We get along well with our neighbours and have harmonious relationships.”

(M001: 34 year old woman, poor, married with two children and no work)

“Here in this community, the community people and the neighbours are good.”

(M007: 42 year old woman, poor, with three children and a husband who works)

For some, a reason for locating to the slum was that relatives were already living in the neighbourhood, this accrued benefits in social support:

“*We have so many relatives living around here*. *They can help us whenever it is necessary*, *that is why we came to live here*.”

(SO07: 36 year old woman, poor, married with two children and no work)

The adversity faced within the slum appeared to strengthen social bonds. In contrast to this, some women shared how those outside the slum discriminated against them:

“We have been living here for seven years, and while we are living here, people from other communities discriminate against us as slum dwellers, we are like refugees. They treat us like slaves.”

(S010: 40 year old woman, better-off, with two children, married and working)

One factor that was identified in both slums by some participants, but not all, was the fact that they did not have to pay rent in the slum, reducing the impact of one of the socio-economic, structural determinants of health. Payments had clearly been crippling for households living in rented accommodation, whereas the slum offered a much cheaper option.

“We don’t have to pay rent. When we were living in Balaju, the landlord asked for the rent within 15 days. It is pleasant living here. It feels like heaven. It’s not bad.”

(M003: 70 year old woman, poor, no children a husband who works)

Slum A had the further advantage of an NGO primary health care clinic and a primary school. This was in clear contrast to their lives outside of the slum.

“The children are admitted in a school. The elder one studies in class five and the younger one is in four. We don't have to pay rent. So, it's very good.”

(M002: 38 year old woman, poor, married with two children and working)

There was some indication from the interviews that the urban area offered a new kind of freedom for women that was not available in their home area. Such a transformation could lessen the impact of this structural determinant of women’s health. This sentiment was only elaborated by one participant originally from the Terai plains, where women’s empowerment indicators are significantly lower than elsewhere in Nepal [22].

“I have my own home back there, but it is divided among the brothers. They do not give me my property because they feel I might marry again. What can I do? I have to take care of my children. In Terai we are not allowed to go out of the house but in the city it is easy to rear children.”

(M010: 30 year old woman, poor, single with two children and working)

### Interventions

During the participatory workshops, after discussing the health problems and risks associated with their kitchens and homes, the women identified small changes that they felt they could implement themselves to reduce these risks.

These were: i) To improve respiratory health: improve ventilation and placement of stove; ii) To avoid gastro-intestinal diseases: Cover leftovers and heat before eating, put food out of reach of children, keep utensils clean, cover drinking water pot, cover dustbin, burn waste when possible, put bleach in the waste and purify water; iii) To avoid accidents and burn injuries: Close gas regulator after cooking, check pipes and regulator for leaks, Keep children away from the stove, Keep knives out of reach of children

This process of brainstorming These simple solutions allowed the women to swap ideas and tips on ways to reduce the risks. There were clearly different levels of knowledge among the women on ways to address these risks and this sharing process was in-itself valuable.

The women also highlighted how they felt the government should manage waste, provide drinking water through more regular tanker supplies and water filters and declare the area a defecation free zone and enforce this rule.

## Discussion

This study brings an in-depth analysis, from the perspective of slum-dwelling women, of the proximal, intermediary and wider structural determinants of health [23,24] and how they impact on health. While our initial protocol focused on the kitchen, it soon became apparent during early interviews that for the women, the cramped slum conditions meant that the concept of a kitchen as a separate room was inappropriate. Kitchen-related activities happened throughout the dwelling and in its close vicinity. Further, the wider environment of the slum frequently intruded on their kitchen-related practices and their health.

### Main health conditions identified by the women

The main health conditions identified by the women were those frequently associated with the poor conditions they were living in and reflecting the so-called ‘traditional environmental risks’ of poor hygiene and sanitation and the use of open fires–namely respiratory disease, gastrointestinal disease and burn injuries. Women demonstrated an understanding of the causes for respiratory and eye problems (smoke from open fires) and for burn injuries (flames and hot liquids/foods). Few women actually associated their lack of access to clean water or sanitation as a risk for gastro-intestinal disease.

### Recognition of Intermediary and Structural Determinants

The women in our study clearly linked the intermediary determinants of health–the psychosocial, material and behavioural–to the common health conditions they and their families’ suffered. For example, women identified poor ventilation, cooking on open fires, over-crowding, lack of adequate child supervision as contributing to the health risks, and linked these hazards with their low household income. Women were also clear on the seasonal dimension to risks. The extent to which the women articulated the stress they experienced and clearly linked this to health conditions such as heart disease as well as contributing to other risk behaviours such as smoking was an important finding of the study. This also concurs with recent evidence from Mumbai slums where an association has been found between household income, poverty-related factors, slum conditions and common mental health problems [25].

While not all women explicitly identified the wider structural determinants of their vulnerability to ill health, these determinants were evident throughout the analysis of the data. Household wealth inevitably interacted with risk. Better-off households were able to afford gas stoves, adequate storage of food stuffs, have water filters or were able to boil water. The poorer households were much more likely to use biomass for cooking, drink water straight from the jar, have limited space in their homes to separate children from fires or dangerous utensils. These findings reflect previous research in slum areas that identify the heterogeneous nature of slums [26]. The characteristics of our participants, as highlighted in Table 1, show clear associations with marital status, number of children, employment and levels of wealth. Those without a husband appear to be facing multiple challenges in working and finding sufficient means to provide for their children. Disability is an added element which appears to keep households firmly in poverty.

### Interplay between determinants

There is a need to recognise the interplay between protective determinants and risk factors. For example while building wood fires for warmth in winter may increase exposure to smoke and burn injuries, they also act as point for social gatherings. The strong social capital that was apparent for some participants was a clear benefit of living in the slum and may well provide a form of protection from risk factors such as stress.

### Intervention Development

Understanding the intermediate and structural determinants allows us to identify which areas are likely to be the most effective to focus on in interventions. For example only focusing on the behavioural aspects–through education or behaviour change techniques—may be of limited value when material or structural determinants are the stronger influences in driving risk. For example, women were well aware of the risks of smoke and burns from chulas, but are unable to switch to using gas due to their low income. No amount of education will change this. Recent literature on improved cook stoves highlights the limited success of these interventions particularly in terms of uptake and sustainable use [27]. In the urban area where gas is clearly the aspiration, interventions to improve income and affordability of LPG are likely to be more effective.

Identifying which determinants operate at household level and which at community level is also key to effective intervention development. For example, better off households had water filters, however these are of little value when communal water sources are contaminated and toilet facilities unavailable. While most participants, except the better-educated, did not identified the link between contaminated water and poor sanitation with gastro-intestinal infections and educational intervention could be of value, however combining this with sanitation improvement at community level would any intervention in this area clearly needs to be at community level as well as household level.

A further important insight from the study is the observation from several participants that while conditions in the informal slum settlement were harsh, renting a room in an established neighbourhood could be worse. Being able to live rent free led one woman to comment that the slum was like ‘heaven’ compared to living in rented accommodation. Those renting in areas that are not such obvious slum informal settlements may be driven deeper into poverty through rents that constitute a high proportion of their income. While such homes still clearly fall within the UN-Habitat definition of a slum, they are not so visible, often co-existing within ‘wealthier’ long established communities. These hidden poor are clearly at risk and may be overlooked by interventions targeting the urban poor.

### Limitations

While we were keen to identify a range of different types of household for our interviews our means of doing this involved the use of community gatekeepers, often clinic staff. This may have meant that certain participants who did not attend the clinic or were not known to the staff may have been overlooked. To overcome this, the researchers explained clearly the purpose of our study and our aim to speak to a range of households by caste, ethnicity, marital status, number of children and relative wealth. Encouragingly, our resulting sample did contain a range of participants across these categories. We were fairly unsuccessful in collecting any consistent information on caste. Clearly this is an important issue of difference within Nepalese society. However, the challenges experienced by the researchers in soliciting any information on individual’s caste reflect the sensitivities associated with this categorisation and limited the generation of any evidence in this area.

A further limitation is the categorisation of wealth. As a team we have attempted to do this based on the researchers’ observations and the photos taken by the women. Finally, two of our research team were new to qualitative research. We conducted several training sessions and on-going support to build their skills in this area. However, some of their earlier interviews were often a little stilted with brief answers. By the final interviews, they were able to build rapport and ask open-ended questions and the quality and depth of the information collected improved. This approach to building capacity for qualitative research was successful and these benefits are to be weighed against the possible limitations on data quality.

A strength of the study was our collection of data in two different types of slum community, one well-establish slum with relatively better-off residents and one more recent development with poorer residents. It should also be noted that, like most slum communities in Kathmandu the settings chosen are along the banks of rivers through the city and are their residents are at similar risk of eviction to other slum areas. These factors all enhance the transferability of our qualitative findings within Kathmandu and should be considered when applying our findings to slum communities elsewhere.

## Conclusions

Women living in slums are very aware of the intermediary determinants–material, behavioural and psycho-social, that increase their vulnerability to ill health. They are also able to identify protective factors, particularly social capital. While they may not explicitly identify the wider social determinants when talking about the issues they face, these are evident. It is only by understanding the determinants at all levels, not just the behavioural, that we will be able to identify appropriate interventions.
